# Up-regulation of Serum MiR-130b-3p Level is Associated with Renal Damage in Early Lupus Nephritis

**DOI:** 10.1038/srep12644

**Published:** 2015-08-28

**Authors:** Wanpeng Wang, Shan Mou, Ling Wang, Minfang Zhang, Xinghua Shao, Wei Fang, Renhua Lu, Chaojun Qi, Zhuping Fan, Qin Cao, Qin Wang, Yan Fang, Zhaohui Ni

**Affiliations:** 1 Department of Nephrology, Renji Hospital, School of Medicine, Shanghai Jiaotong University; 2 Department of Health Care center, Renji Hospital, School of Medicine, Shanghai Jiaotong University

## Abstract

Systemic lupus erythematosus (SLE) is a common but severe autoimmune systemic inflammatory disease. Lupus nephritis (LN) is a serious complication of SLE,affecting up to 70% of SLE patients. Circulating microRNAs (miRNA) are emerging as biomarkers for pathological conditions and play significant roles in intercellular communication. In present research, serum samples from healthy control, early and late stage LN patients were used to analyze the expression profile of miRNAs by microarray. Subsequent study demonstrated that miR-130b-3p in serum of patients with early stage LN were significantly up-regulated when compared with healthy controls. In addition,we have also observed that the expression of a large amount of circulating microRNAs significantly decreased in patients with late stage LN. The further analysis found that the expression of serum miR-130b-3p was positively correlated with 24-hour proteinuria and renal chronicity index in patients with early stage LN.Transfection of renal tubular cellline(HK-2)with miR-130b-3p mimics can promote epithelial-mesenchymal transition (EMT). The opposite effects were observed when transfected with miR-130b-3p inhibitors. MiR-130b-3p negatively regulated ERBB2IP expression by directly targeting the 3′-UTR of ERBB2IP The circulating miR-130b-3p might serve as a biomarker and play an important role in renal damage in early stage LN patients.

Systemic lupus erythematosus (SLE) is a common but severe autoimmune disease characterized by the presence of autoantibodies against several self-antigens, which causes serious injury to various organs or systems^1^,^2^,^3^. Lupus nephritis (LN) is a common and serious complication that is often associated with a poor long-term prognosis; up to 70% of SLE patients are affected of LN and about 10–30% of which will progress to end-stage renal failure (ESRD)^4^,^5^,^6^ Therefore, early diagnosis and prompt treatment may significantly improve prognosis.

MicroRNAs (miRNAs) are short non-coding RNA molecules that inhibit gene expression through incomplete base pairing with the 3′-untranslated region (3′-UTR) of target mRNAs^7^,^8^. The miRNA system is conserved from worms to mammals and contributes to the regulation of a wide variety of cellular functions^9^. Many studies indicated that inappropriate expression of miRNA is seen in a wide variety of autoimmunity disorders. Thus, the expression profiles of miRNAs are effective for classification of human diseases^10^,^11^,^12^.

Prior researchers have reported the presence of circulating miRNAs in human serum, plasma, urine, and other body fluids^13^,^14^,^15^. These miRNAs are not affected by endogenous RNases in the plasma^16^,^17^. In addition, circulating miRNAs significantly altered in disease conditions than in healthy individuals^13^,^18^. The characteristics of circulating miRNAs have established their promising value as biomarkers in physiological and pathological conditions. The altered expression of miRNAs is also found in serum and urine from lupus patients and is involved in the development of lupus nephritis^15^,^19^,^20^. On account of the extremely complex pathogenesis of immune dysfunction in SLE, it is possible that many more miRNAs may be involved in its immunopathogenesis. Therefore, we attempted to reveal the changes of miRNAs in serum during different stages of CKD caused by LN utilizing microarray technology, and investigated their correlation with renal damage and the possible molecular mechanism involved in early stage LN.

## Materials and Methods

### Study Design and Recruitment

A total of 96 serum samples, including 60 LN patients with different stages of CKD and 36 healthy controls, were included in this study. Blood samples from 4 early stage LN patients(CKD 1–3 stage), 4 late stage LNpatients(CKD 4–5 stage) and 4 healthy controls were used to analyze the expression profile of circulating miRNA. Another 52 LN patients (including 40 patients with early stage LN and 12 patients with late stage LN), and 32 age- and sex-matched healthy control were recruited for the validation group. All LN patients were confirmed by biopsy and fulfilledthe 1982 American College of Rheumatology (ACR) revised criteria for SLE. The study was approved by the Ethics Committee of Renji Hospital. All participating subjects gave written consent according to the Declaration of Helsinki. All serum samples were collected from May to October 2013. 40 patients with early stage LN were diagnosed between February 2012 and October 2013 by biopsy. Modification of Diet in Renal Disease (MDRD) formula was used to estimate glomerular filtration rate (eGFR). Demographic and clinical data of the patients were recorded. Blood samples were collected at least 24 h after the last dose of immunosuppressant in order to minimize drug the influences of the medication.

### Definition of SLE Activity and Renal Histopathology

The disease activity was assessed according to the Systemic Lupus Erythematosus Disease Activity Index (SLEDAI)^21^. For renal involvement, renal SLE Disease Activity Index (SLEDAI) was used to evaluate kidney disease activity. The score included 4 kidney related clinical parameters: hematuria, pyuria, proteinuria, and urinary casts. Scores for the renal SLEDAI can range from 0 to 16. Renal histopathology was classified according to 2003 revised criteria for glomerulonephritis of SLE which was published by International Society of Nephrology/Renal Pathology Society^22^. Activity index (AI) and chronicity index (CI) were scored using a previously reported system involving semi-quantitative scoring of specific biopsy features. Components of AI including: (1) endocapillary hypercellularity, (2) glomerular leukocyte infiltration, (3) cellular crescents, (4) karyorrhexis, (5) fibrinoid necrosis, (6) formation of wire loop, (7) interstitial inflammatory cell infiltration; Components of CI including: (1) glomerular sclerosis, (2) fibrous crescents, (3) interstitial fibrosis, and (4) tubular atrophy.

### Serum miRNA Expression Profiling

First, serum samples (n = 4 from each group) from healthy control, early stage LN patients (eGFR > 30 mL/min/1.73 cm^2^ CKD 1–3 stage) and late stage (eGFR < 30 mL/min/1.73 cm^2^ CKD 4–5 stage) LN patients were used to analyze the expression profile of human miRNAs by Human Serum & Plasma miScriptmiRNA PCR Array (MIHS-3106Z, Qiagen, USA), which profiles the detectable expression of 372 miRNAs and is differentially expressed in serum, plasma and other body fluids. ALL of these 8 screening LN patients were classified as Class IV according to ISN/RPS 2003 classification. In brief, RNA was reverse transcribed to cDNA using miScript Reverse Transcription kit (Qiagen,USA) according to the manufacturer’s instructions. Real-time qPCR was performed using miScript SYBR Green PCR kit (Qiagen,USA) with the manufacturer provided miScript Universal primer. Microarray data were analyzed using free web software (http://pcrdataanalysis.sabiosciences.com/mirna/arrayanalysis.php) and all 2^−∆∆Ct^ fold change were calculated automatically.

### MiRNA specific Quantitative Real-Time RT-PCR

Another 52 LN patients (including 40 patients with early stage LN and 12 patients with early stage LN), and 32 age- and sex-matched healthy control were recruited for the validation group. Total RNA was isolated from 200 μL of serum by miRNeasy Serum/Plasma Kit (Qiagen, USA), according to the manufacturer’s instructions. Synthetic spiked-in *C.elegans* miR-39 was added to the serum prior to RNA extraction as an internal control. The RNA pellet was resuspended in 12 μL RNase-free H_2_O. Nanodrop 2000 was used for Detecting RNA concentration. TaqMan miRNA reverse transcription kit (Applied Biosystems, USA) was used for reverse transcription. In brief, 1.67 μL total RNA was mixed with 1 μL specific primers, 0.05 μLdNTPs, 0.5 μL 10× reverse transcription buffer, 0.33 μL MultiScribe Reverse Transcriptase, 0.05 μL RNase inhibitor; and made up to 5 μL with H_2_O. Reverse transcription was performed under the following conditions: 16 °C for 30 min, 42 °C for 30 min, and 85 °C for 5 min. The resulting cDNA was stored in −80 °C until use. Real-time PCR was performed using Master Mix II and no UNG (Applied Biosystems, USA) and Lightcycler 480 Real Time PCR System.

### Cell Culture and MiRNA Transfection

Human renal tubular epithelial cell line HK-2 was obtained from The Global Biosource Center (ATCC). The cells were maintained in DMEM/F12 (GIBCO,USA), supplemented with 10% Fetal Bovine Serum (FBS) (GIBCO,USA). Cells were grown to confluence and serum deprived for 24 hours before transfection with miR-130b-3p mimics or inhibitors or correspondent controls (Sunbio, Shanghai) using Lipofectamine^®^RNAiMAX Transfection Reagent (Applied Biosystems, USA). The medium was replaced with DMEM/F12 culture medium containing 2% reduced FBS at 6h post transfection. Then, HK-2 cells were stimulated with 10 ng/mL recombinant TGF-β1 (PeproTech, USA) for 72 hours.

### mRNA analysis of the HK-2 cells

Total RNA from the transfected HK-2 cell line was extracted using Trizol reagent (TaKaRa Bio, Japan). The expression levels of E-cadherin (E-cad) and α-smooth muscle Actin(α-SMA), ERBB2IP mRNA were quantified by real-time PCR using the RT-PCR and SYBR Green kit (TaKaRa Bio, Japan) on a LightCycler 480 Real Time PCR System. The primers for E-cad were 5′-GAGTGCCAACTGGACCATTCAGTA-3′ (forward) and 5′-AGTCACCCACCTCTAAGGCCATC-3′ (reverse). The primers for α-SMA were 5′-ATAGAACATGGCATCATCACCAAC-3′ (forward) and 5′-GGGCAACACGAAGCTCATTGTA-3′ (reverse). The primers for ERBB2IP were 5′-CCCTTCATTGTGGGAGGAAC-3′ (forward) and 5′-CTCATCTGGGTATGGTGTTGG -3′ (reverse). Conditions for the quantitative PCR were 37 °C for 15 min and 85 °C for 5 s for RT, followed by 40 cycles of 95 °C for 5 s and 60 °C for 30 s. Expression of GADPH mRNA was used as endogenous control for data normalization. For miRNA, expression of U6 small RNA was used as an endogenous control for data normalization.

### Western Immunoblot Analysis for α-SMA, E-cadherin and ERBB2IP

Seventy-two hours after transfection with miR-130b-3p mimics or inhibitors, the cells were lysed with RIPA buffer (Thermo Fisher Scientific,USA) respectively, and Western blotting was performed using standard procedures. The primary antibodies used for the analysis were rabbit anti-human E-Cadherin Polyclonal antibody (1:300,abcam); α-SMA rabbit polyclonal antibody mAb (1:300,abcam), sheep polyclonal ERBB2IP antibody (1:200,R & D Systems), and GAPDH rabbit polyclonal GAPDH rabbit polyclonal (1:1000,Sigma). Goat anti-rabbit and goat anti-sheep Horseradish peroxidase-conjugated (HRP) antibodies (1:2000, Jakeson) were used as secondary antibodies. The immunelabeled proteins were detected by enhanced chemiluminescense (Pierce). The density of bands was analyzed by Gel-Pro Analyzer analysis software.

### Luciferase Activity Assay

The PCR fragment of ERBB2IP 3′-UTR of HK-2 cells was digested with Xho I and Not I and cloned into psiCheck-2 (Promega, USA). The primers for 3′-UTR of ERBB2IP were 5′-AGGCGATCGCTCGAGGCACTGTGGACAAAAAAAGC-3′(forward) and 5′-ATTCCCGGG CTCGAGTCCTCAAAAAATTTAATTATTTTATTCA-3′(reverse). The DNA order of report vectors was confirmed by DNA sequencing. For the reporter assays, 293T cells was transiently transfected with reporter plasmids (500 ng) and negative control mimics (NC mimics), wild-type miR-130b-3p mimics or mutant miR-130b-3p mimics (Sunbio Corporation, Shanghai) using Lipofectamine 2000 At 48 h post transfection, cells were harvested for analysis. Luciferase activity was determined by using a Promega luciferase assay system. All experiments were performed in triplicate and means were determined.

### Statistical Analysis

Data were analyzed by non-parametric tests using Mann-Whitney U-test for two nonparametric groups. Receiver operating characteristic (ROC) curves were constructed and the area under the curve (AUC) was calculated to evaluate specificity and sensitivity of predictive value or feasibility of using serum miRNA as a biomarker for LN. Correlations were determined by the Spearman rank correlation coefficient. All tests were 2-sided and p-value less than 0.05 were considered statistically. All statistical analyses were performed and graphs were generated using GraphPad Prism 5.0 (GraphPad Software Inc, California).

## Result

### Circulating miRNAsprofile in different stage LN patients

**Figure 1:**
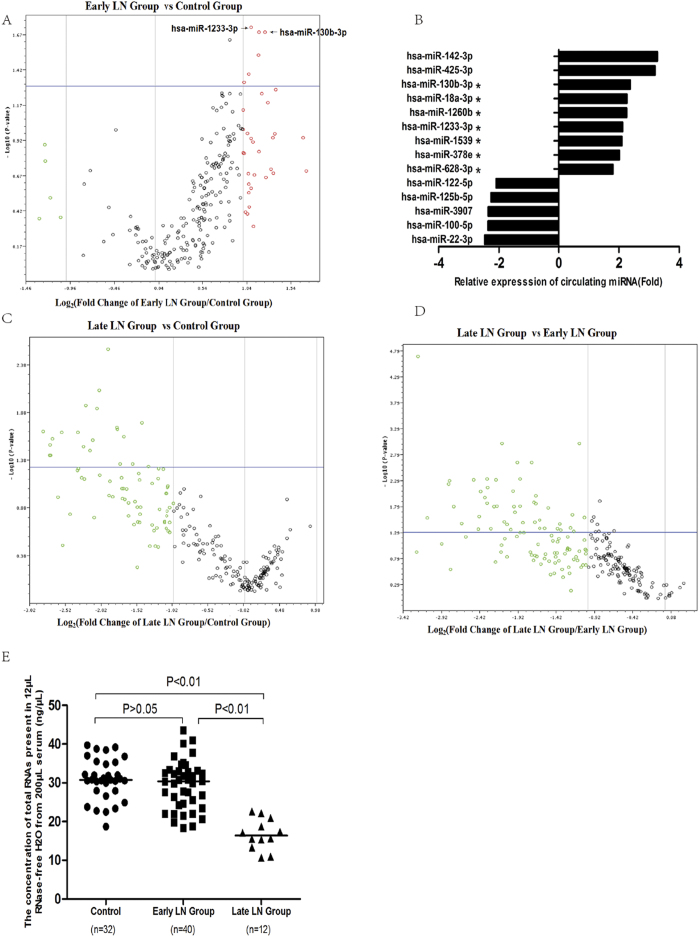
erum miRNA expression in patients with lupus nephritis (LN). (**A**) Serum miRNAs profile from 4 early stage LN patients (eGFR > 30 mL/min/1.73 cm^2^) versus 4 healthy controls. (**B**) The fold of serum miRNA changes of early stage LN versus healthy controls. (**C**) SerummiRNAsprofile from 4 late stage LN patients (eGFR < 30 mL/min/1.73 cm^2^, CKD 4–5 stage) versus 4 healthy controls. (**D**) SerummiRNAs profile from 4 late stage versus 4 early stage LN patients. (**E**) The concentration of total RNAs present in 12 μL Rnase water from 200 μL serum of the validation group. The vertical line represented the changing fold equal to –2 (down-regulation), 1, 2 (up-regulation and invisible in Fig. 1D). The plots above the horizontal line represent the miRNAs with P values less than 0.05. *P < 0.05 **P < 0.01.

To identify miRNAs that were differentially expressed in serum of LN patient, we analyzed 372 human miRNAs in serum from 4 healthy controls, 4 early stage LN patients (eGFR > 30 mL/min/1.73 cm^2^, CKD 1–3) and 4 late stage LN patients (eGFR < 30 mL/min/1.73 cm^2^, CKD 4–5). We found that the expressions of seven miRNAs, including miR-130b-3p, miR-1233-3p, miR-18a-3p, miR-628-3p, miR-1260b, miR-1539 and miR-378e were increased in early stage LN patients compared to healthy controls (P < 0.05) (Fig. 1A,B). On the other hand, the expression level of 75 circulating miRNAs were found decreased more than 2 times, and 21 miRNAs down-regulated significantly (P < 0.05) in late stage LN patients compared to healthy controls; the expression level of 100 circulating miRNAs in detected were found decreased more than 2 times, and 53 miRNAs down-regulated significantly (P < 0.05) in late stage LN patients compared to early stage LN group. There was no miRNAs were found up-regulated more than 2 times or increased significantly (P < 0.05) in late stage LN group compared to healthy controls or early stage LN patients (Fig. 1C,D, Supplementary Table 1a–c). The results have a implication that circulating miRNA may reduced in patients with severe chronic renal failure. This finding was also confirmed by total RNA concentration analysis of the validation group (Fig. 1E).

### Serum MiRNA-130b-3p differentially expressed in LN Patients

**Figure 2:**
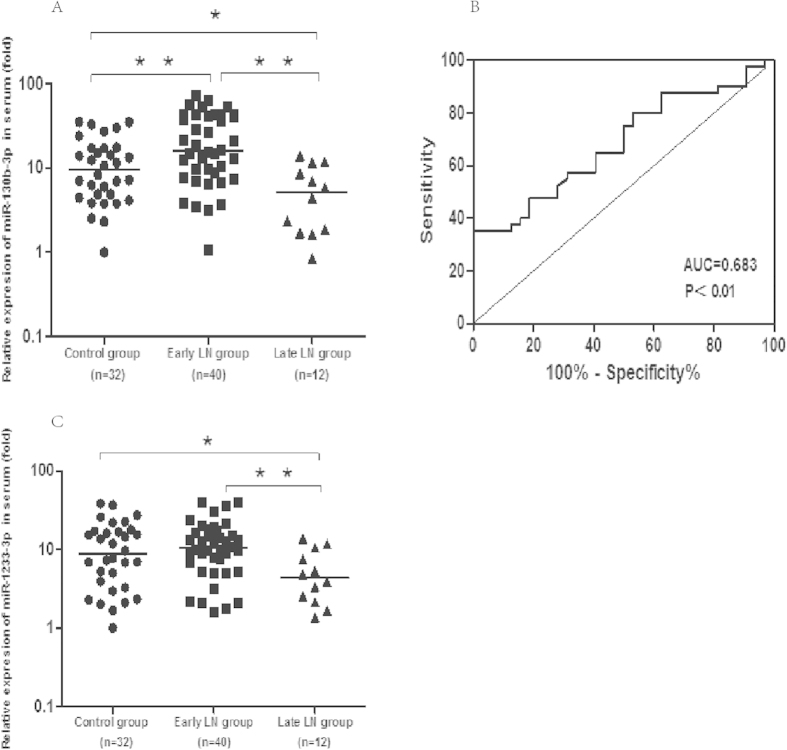
Expression of miR-130b-3p and miR-1233-3p in validation group. (**A**) Serum miR-130b-3p indifferent stage of LN patients. (**B**) Receiver operating characteristic (ROC) curves with corresponding area under the ROC curve (AUC) for miR-130b-3p in discriminating LN patients with early stage CKD (stage1–3) from healthy control. (**C**) Serum miR-1233-3p indifferent stage of LN patients. The line indicates the median value per group. Fold regulation is expressed as RQ based on 2^−∆∆Ct^ *P < 0.05 **P < 0.01

Another 52 LN patients and 32 age- and sex-matched healthy control were recruited for the validation group (Table 1). Two miRNAs, miR-130b-3p, miR-1233-3p, which increased the most and have the lowest p value in early stage LN in the screening group were selected to be validated. The results showed that the level of miR-130b-3p were significantly higher in early stage LN patients compared to healthy controls [IQR 16.2 (8.7, 42.7) *vs* 9.6 (4.8, 17.4), p < 0.01)], and decreased significantly in late stage LN group (Fig. 2A). The miR-1233-3p was not significantly changed between controls and early stage LN patients [IQR 10.4 (6.4, 16.9) *vs* 8.7 (3.8, 16.9) p > 0.05],but significantly down-regulated in late stage LN group (Fig. 2C).The area under the ROC curve (AUC) for miR-130b-3p in predicting early stage LN from healthy control is 0.683 ± 0.062 (95%CI = 0.561–0.805, P < 0.01) (Fig. 2B).

### Serum MiR-130b-3p was not affected by SLE activity but correlated with renal damage

**Figure 3:**
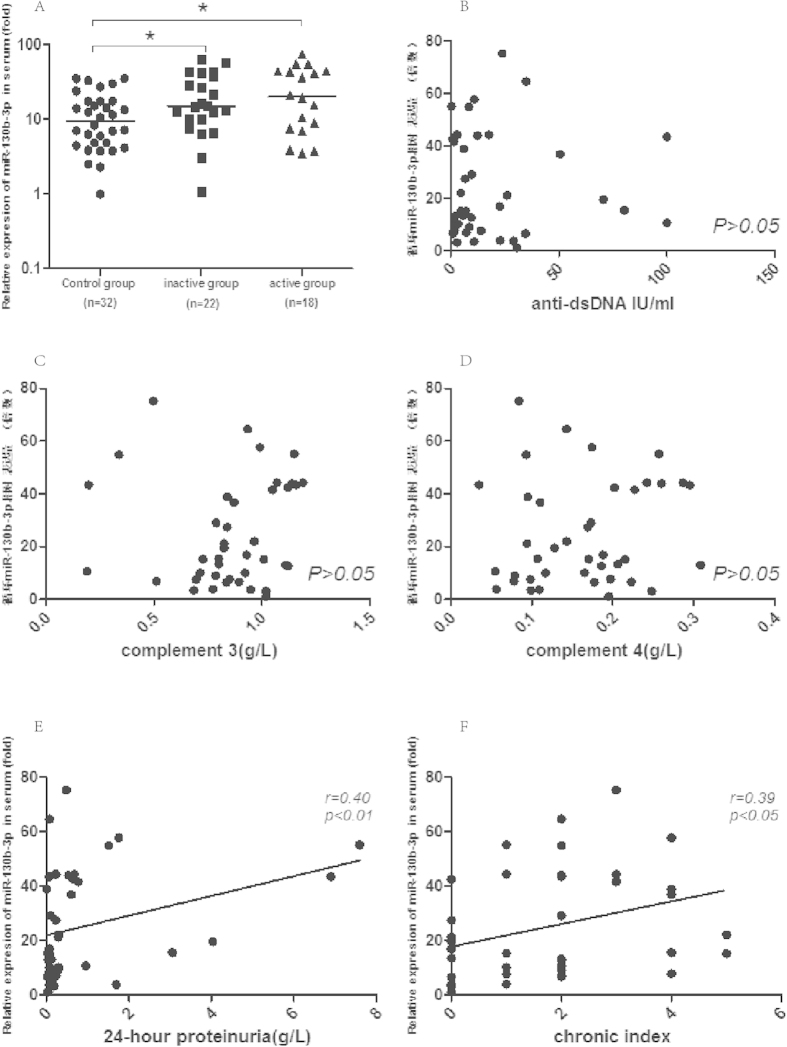
The association of serum MiR-130b-3p and SLE activity and renal damage. (**A**) Altered expression of serum miR-130b-3p in early stage LN patients with or without SLE activity. The line indicates the median value per group. Fold regulation is expressed as RQ based on 2^−∆∆Ct.^; The correlation between serum miR-130b-3p and (**B**) anti-dsDNA, (**C**) C3, (**D**) C4, (**E**) 24-hour urine proteinor, (**F**) renal chronicity index in early stage LN.

To determine whether serum miR-130b-3p correlates with SLE activity, early stage LN patients were divided into inactive and active groups based on the SLE disease activity index (SLEDAI). There are 22 patients whose SLEDAI score <9 belong to inactive group and 18 patients whose SLEDAI ≥ 9 to active group^23^. The anti-double stranded DNA (anti- dsDNA), complement 3(C3), complement 4(C4), 24-hours urinary proteinuria, sedimentation rate (ESR) were significantly different between active and inactive group (Table 2). The miR-130b-3p expression was significantly upregulated both in inactive and active patients as compared to healthy controls [IQR 15.2 (10.1, 36.4) vs9.6 (4.8, 17.4), P < 0.05; and 20.4 (8.0, 43.8) vs9.6 (4.8, 17.4), *P* < 0.05]. In spite of a higher median, there were no statistical significance found between active group and inactive group (P > 0.05) (Fig. 3A). The association of serum miR-130b-3p and various disease activity parameters were found not significant correlated such as anti-dsDNA (Fig. 3B), C3 (Fig. 3C), C4 (Fig. 3D), etc(details in Table 3). The data showed that the expression level of serum miR-130b-3p may not be affected by SLE disease activity.

The association analysis showed that the expression of serum miR-130b-3p was significantly and positively correlated with 24-hour proteinuria (Fig. 3E) and chronicity index (Fig. 3F). Our data also showed that serum miR-130b-3p was well correlated with serum triglyceride level (Table 3) which in accordance with the report by Wang *et al.*^24^.

### Serum MiR-130b-3p was correlated with EMT of renal tubular cells

**Figure 4:**
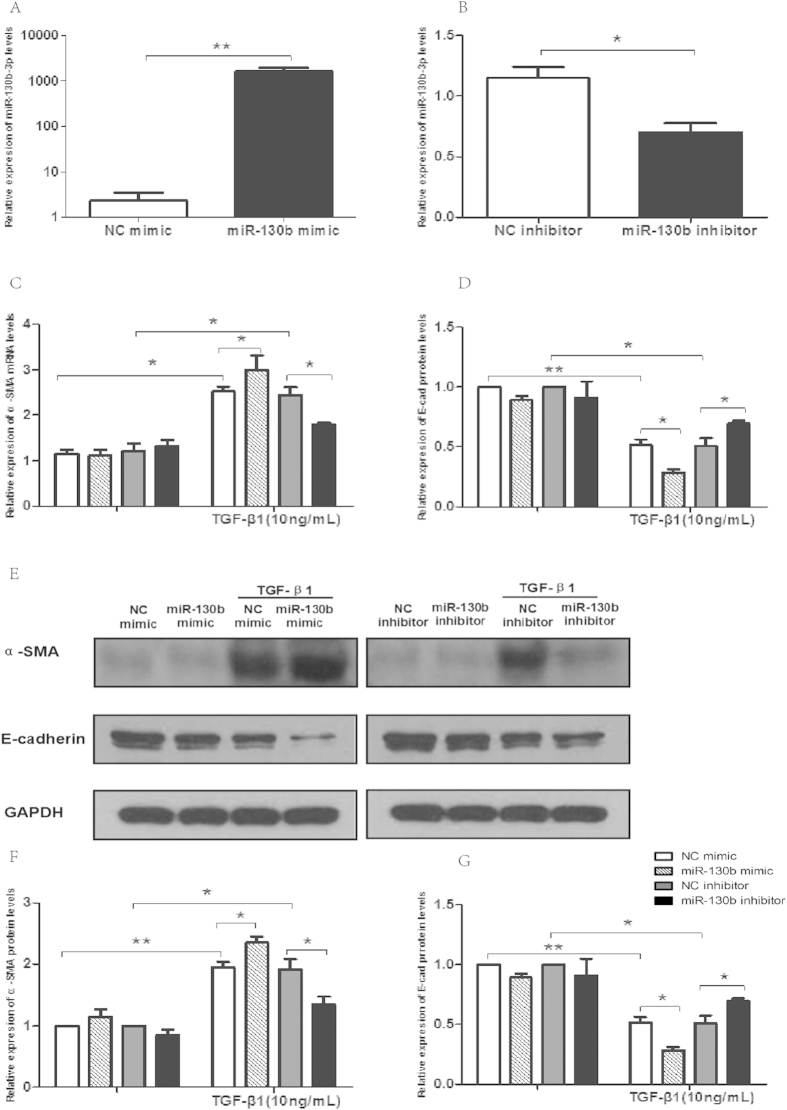
The role of miR-130b-3p in renal tubular cells. (**A**) The expression of miR-130b-3p was significantly upregulated in HK-2 cells post transfecting with miR-130b-3p mimics than with miR-control at a 30nM concentration. (**B**) MiR-130b-3p expression in HK-2 cells was determined by quantitative RT-PCR 24 h post transfection of miR-130b inhibitors. (**C**,**D**) Transfection of HK-2 cells with miR-130b-3p mimics resulted in significantly increased mRNA expression of α-smooth muscle actin (α-SMA) and decreased mRNA expression of E-cadherin (E-cad) compared to NC mimics in the presence of TGF-β1 (10 ng/ml) for 72 h; Inhibition of miR-130b-3p partially reversed TGF-β1-induced EMT. (G-F) HK2 cell’s expression of E-cadherin or α-SMA protein after transfection of miR-130b-3p mimics or inhibitors and correspondent controls when in the absence or presence of TGF-β1 (10 ng/ml). All results are expressed as mean ± SD. for three independent experiments. *p < 0.05, **p < 0.01

Since circulating miR-130b-3p expression significantly upregulated in LN patients and positively correlated with renal damage and chronicity index, we hypothesized that the increasing serum miR-130b-3p may correlated with the pathogenesis of tubulointerstitial lesions of lupus nephritis. To further understand the role of miR-130b-3p in renal tubular cells, HK-2 cells were transfected by miR-130b-3p mimics or inhibitors. The expression of miR-130b-3p was significantly upregulated in HK-2 cells transfected by miR-130b-3p mimics and significantly down-regulated transfected by inhibitors compared to controls (Fig. 4A,B). Transfection of HK-2 cells with miR-130b-3p mimics resulted in significantly increased expression of α-smooth muscle actin(α-SMA) and decreased expression of E-cadherin (E-cad) in the presence of TGF-β1 (10 ng/ml) both on RNA and protein level. Furthermore, inhibition of miR-130b-3p partially reversed TGF-β1-induced EMT and fibrosis (Fig. 4C–G).

### MiR-130b-3p promote EMT by Targeting ERBB2IP

**Figure 5:**
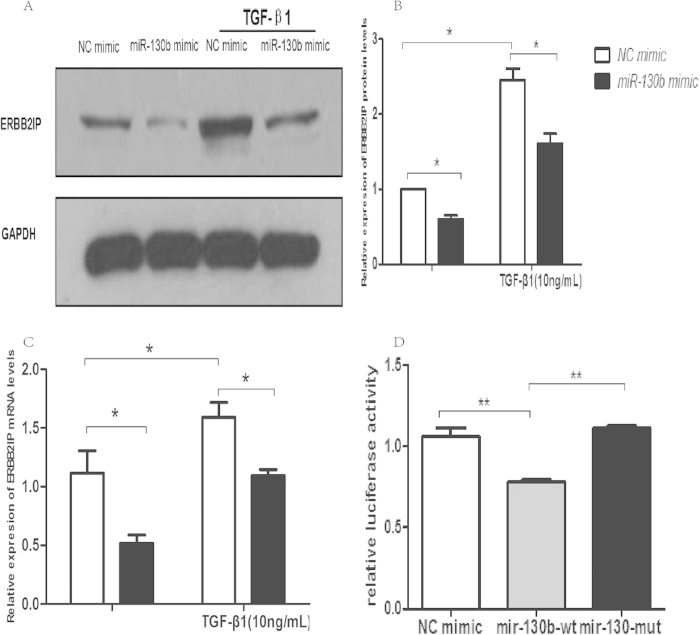
miR-130b-3p promotes TGF-β1-induced EMT in renal tubular epithelial cells by targeting ERBB2IP. (**A**,**B**) Transfection of HK-2 cells with miR-130b-3p mimics resulted in significantly decreased protein expression of ERBB2IP compared to NC mimics both in the absence and presence of TGF-β1 (10 ng/ml) for 72 h. (**C**). Transfection of HK-2 cells with miR-130b-3P mimics resulted in significantly decreased mRNA expression of ERBB2IP in the absence and presence of TGF-β1 (10 ng/ml) for 72 h (**D**) The effects of mimics negative controls (NC-mimics), wild-type miR-130b-3p mimics(miR-130b-wt) and miR-130b-mut on luciferase activity in 293T cells after transfection with 500 ng psiCHECK-2 reporter plasmids. All results are expressed as mean ± SD. for three independent experiments. *p < 0.05, **p < 0.01.

Nine microRNA target prediction programs (DIANAmT, miRanda, miRDB, miRWalk,RNAhybrid, PICTAR, PITA, RNA22 and Targetscan, the details in Supplementary Table 2) were used to identify the potential targets of miR-130b-3p in human, mouse, and rat transcripts. Since the predictions from each program were different owing to different algorithms to predict microRNA targets, only targets predicted by all these programs from human, mouse, and rat transcripts were considered. Using this approach, we identified 7 potential targets, including MED12L, PHF3, NEUROD1, INOC1, ERBB2IP, CHD9, and ARHGAP12. We selected ERBB2IP for further study, as ERBB2IP is reported to have a role in regulating EMT in renal tubular cells^25^. Consistent with previous reports, our Western blotting and real-time PCR data showed that TGF-β1 upregulated ERBB2IP expression in renal tubular cells. After transfected by miR-130b-3p mimics both in the absence and presence of TGF-β1 (Fig. 5A–C) showed significantly decreasing ofERBB2IP mRNA and protein. To confirm whether the effect of miR-130b-3p was a direct effect, the luciferase-reporter plasmids vectors containing DNA segments of ERBB2IP 3′-UTR were constructed then confirmed by DNA sequencing. Following co-transfection of human 293T with the constructs and one of the mimics: negative control, wild-type miR-130b-3p, mutant miR-130b-3p, luciferase activity was measured in each case. The results showed that miR-130b-3p induced a decrease of luciferase activity compared to negative control and mutant miR-130b-3p, but mutant miR-130b-3p had similar effect compared to negative control (Fig. 5D).

## Discussion

One of the many advantages of using miRNAs as disease biomarkers is the availability of highly sensitive PCR detection methods and their low complexity compared with protein biomarkers^26^. Unlike large numbers of mRNAs, a small group of miRNAs or even one specific miRNA might be sufficient to differentiate patients from healthy individuals^27^. In addition, pathogenic miRNAs may be able to detect early LN onset in SLE patients before clinical and pathological findings arise. To the best of our knowledge, the present study is the first one to prospectively analyze 372 serum miRNAs in LN patients with different CKD stages by microarray.

One implications of this work by microarray is that, compared with mild renal impairment or normal renal function patients, the circulating miRNAs in renal failure patients may be significantly decreased. The mechanism may related with marked accumulation of RNase in renal failure patients which increased the degradation of circulating miRNAs^28^,^29^,^30^, but it is still unclear. This observation is supported by the fact that the total RNA level was significantly lower and miR-130b-3p and miR-1233-3p were found down regulated respectively in late stage LN of validation group. Smilar results were reported by Neal *et al.*^31^, who detected 5 specific miRNAs in serum of patient with renal failure. But larger sample size and more circulating miRNAs should be studied in the future to confirm the finding. Among seven differentially expressed miRNAs discovered by microarray, serum miR-130b-3p was confirmed up-regulated confirmed by validation group. ROC curves analysis demonstrated that circulating miR-130b-3p may be used as a predictive biomarker for early stage LN. Furthermore, the circulating miR-130b-3p is correlated with proteinuria and renal chronicity index of LN, which suggests that miR-130b-3p may be used as a marker for renal damage of LN at early stage. It will be interesting to further understand the role of miR-130b-3p in the pathogenesis and prognosis of LN.

It is well known that circulating miRNAs can be packaged in microparticles (MPs),include microvesicles(100 nm–1 μm) and exosomes(40–100 μm), which can stably exist in the blood, urine, and other body fluids of patients^17^. Until now, Although there have been no direct evidence show whether circulating miR-130b-3p are able to be taken up by tubular epithelial cell and affect its functions, some studies have pointed out that circulating MPs can mediate cell communication through long-distance targeting via the systemic circulation similar to hormones. This observation is supported by the fact that exosomes extracted from the cultured mesenchymal stem cells were administered systemically to animals and protected them against acute kidney injury (AKI) induced by ischemia/reperfusion(I/R)^32^ and nephrotoxic antibiotics^33^. In particular, the findings reported by Cantaluppi *et al.* suggest that MPs from endothelial progenitor cells (EPCs) protect from I/R injury^34^, and the dye-labeled MPs were detected not only in endothelial but also in tubular epithelial cells 2 hours after tail vein injection of MPs. All those researches suggested that circulating MPs can have remote function or can be transferred to target cells such as tubular epithelial cells. However further researches are needed to illuminate this process. The current study found that when HK-2 cells were transfected by miR-130b-3p mimics or inhibitors, the EMT process of the cells were influenced. Although the current study did not search for the resources of the circulating miR-130b-3p, identifying of its up-regulation may provide new insight into disease pathogenesis and novel therapeutic targets for LN.

Previous studies have shown that miR-130b-3p plays an important role in multiple carcinomas. Yip *et al.* showed that the down-regulated miR-130b was highly correlated with the aggressive phenotype of papillary thyroid carcinoma^35^. However, the other results demonstrated that miR-130b was significantly upregulated and acts as a tumor promoter^36^. Colangelo *et al.* demonstrated that miR-130b up-regulation exhibits poor patient prognosis, and enhanced EMT and angiogenesis^37^. In this stdudy, after miR-130b-p mimics were transfected to HK-2 cells, epithelial-mesenchymal transition- (EMT-) like changes in HK-2 cells were promoted under stimulation of TGF-β1, characterized by decreased E-cadherin expression and increased α-smooth muscle actin (α-SMA). On the contrary, transfection of miR-130b-3p inhibitors can prevent TGF-β1 induced EMT by increasing E-cadherin expression and decreasing expression of α-SMA.

The most interesting finding of the present study is that ERBB2IP were demonstrated as apotential target of miR-130b-3p. ERBB2IP is also known as ERBIN or ERBB2/HER2 interacting protein. Previous studies have reported that the PDZ domain of EIBB2P can interact with various proteins involved in cell–cell or cell–matrix interaction^38^, such as ErbB2, Nod2^39^, p120 protein, and β4-integrin. It participates in the regulation multitudes of cellular functions. ERBB2IP has been reported as a negative regulator of Smads and ERK which are important TGF-β-induced signaling pathways and Erbin can sequester Smad2/Smad3 complex from their association with Smad4 and thereby negatively modulates TGF-β-dependent cell growth^40^. Moreover, Zhou *et al.* revealed that ERBB2IP is a negative feedback molecule induced by TGF-β1 and inhibits TGF-β1-induced EMT in renal tubular cells^25^. Our findings of miR-130b-3p directly targeted ERBB2IP and caused the following pathogenesis such as EMT, It is promising to launch further research in this area.

Recent observations of miR-130b have suggested PGC-1α and PPAR-γ^24^,^41^ as its direct target gene. A series of studies has showed PPAR-γ agonist protects podocytes from injury^42^,^43^. Our results showed that serum miR-130b-3p was positively correlated with 24-hour proteinuria may indicate the same idea. It is worthwhile to further understand the mechanism of miR-130b-3p, proteinuria and podocyte in the future. A well designed study and larger sample size are needed in the future and it will be more promising if other forms of kidney diseases included.

## Conclusion

In summary, we have demonstrated that the serum miR-130b-3p correlated with LN and my serve as a biomarker for early stage LN. Through its target gene ERBB2IP, miR-130b-3p participated in the prognosis of LN.

## Additional Information

**How to cite this article**: Wang, W. *et al.* Upregulation of Serum MiR-130b-3p Level is Associated with Renal Damage in Early Lupus Nephritis. *Sci. Rep.* **5**, 12644; doi: 10.1038/srep12644 (2015).

## Supplementary Material

Supplementary Information

## Figures and Tables

**Table 1:** Summary of the validation group

	Control	LN patient withearly stage CKD(eGFR > 30)	LN patient withlate stage CKD(eGFR < 30)
Number, n	32	40	12
aAge, y	42.03 ± 10.38	37.54 ± 12.66	46.27 ± 14.73
Female, n. (%)	30 (93.8%)	38 (95%)	10 (83.3%)
aSerum creatinine(SCr), μmol/L	—	60.45 ± 20.96	877.29 ± 450.65
aeGFR mL/min/1.73 cm^2^	—	114.37 ± 29.18	8.86 ± 8.46
CKD Stage 1, n (eGFR > 90)	—	31	—
CKD Stage 2, n (eGFR 60–89)	—	7	—
CKD Stage 3, n (eGFR 30–59)	—	2	—
CKD Stage4, n(eGFR 15–29)	—	—	2
CKD Stage5, n (eGFR < 15)	—	—	10

Abbreviations: LN: lupus nephritis; eGFR: estimated glomerularfiltration rate; CKD: chronic renal desease. a Values are mean ± SD.

**Table 2:** Clinical and laboratory data of active and non-active LNgroups.

	Inactive group	Active group	P value
**Demographic**			
Number, n	22	18	
Age, y, mean ± SD	37.2 ± 13.4	37.9 ± 12.1	0.870
Female, n. (%)	22 (100%)	16 (88.9%)	0.221
**Biopsy- proven lupus nephritis, n. (%)**			
WHO class III ± V	1(16.0%)	4(6.7%)	0.230
WHO class IV ± V	15 (68.0%)	13(73.3%)	0.781
WHO class V	6(16.0%)	1(20.0%)	0.156
**Clinical and laboratory findings (mean** ± **SD)**			
MAP, mmHg	89.7 ± 6.5	92.1 ± 6.0	0.232
Serum WBC, 10^9^/L	6.34 ± 2.10	6.11 ± 2.50	0.762
Hemoglobin(Hb), g/L	118.3 ± 17.9	121.3 ± 16.2	0.590
Platelets(PLT), 10^9^/L	201.5 ± 66.7	202.3 ± 73.7	0.972
Urinary WBC, cell/HPF	4.01 ± 4.63	9.79 ± 11.86	0.042
Urinary RBC, cell/HPF	2.94 ± 3.4	25.11 ± 59.49	0.088
24 hour proteinuria, g	0.218 ± 0.373	1.662 ± 2.293	0.006
SLEDAI(renal)	2.182 ± 2.683	7.333 ± 4.602	0.000
Anti-dsDNA, IU/ml	9.351 ± 10.898	32.447 ± 33.123	0.004
Serum C3, g/L	0.939 ± 0.131	0.749 ± 0.305	0.012
Serum C4, g/L	0.192 ± 0.051	0.125 ± 0.077	0.002
ESR, mm/h	10.5 ± 7.3	28.6 ± 18.5	0.000
Serum creatinine(SCr), μmol/L	59.36 ± 15.03	61.79 ± 26.92	0.719
eGFR mL/min/1.73cm^2^	112.9 ± 26.7	116.2 ± 32.6	0.724
Blood Urea Nitrogen(BUN),mmol/L	4.84 ± 1.54	5.12 ± 2.24	0.641
Serum Uric Acid (UA) μmol/L	293.0 ± 113.8	347.9 ± 98.5	0.116
Serum albumin (ALB), g/L	39.96 ± 4.87	32.46 ± 6.95	0.000
High density lipoprotein(HDL), mmol/L	1.580 ± 0.296	1.254 ± 0.403	0.005
Low density lipoprotein (LDL), mmol/L	2.224 ± 0.296	3.345 ± 1.219	0.001
Alkaline phosphatase(AKP), U/L	49.0 ± 16.8	55.5 ± 24.0	0.318
ALT U/L	17.5 ± 11.9	15.2 ± 9.6	0.514
AST U/L	17.2 ± 5.2	21.2 ± 23.4	0.446
Triglyceride(TG), mmol/L	1.296 ± 0.404	1.989 ± 0.791	0.001
Total cholesterol (TC), mmol/L	4.201 ± 0.928	5.357 ± 1.380	0.003
Fasting blood-glucose (FBG), mmol/L	4.029 ± 0.603	4.347 ± 0.828	0.168

Abbreviations: MAP: mean artery pressure; ESR: erythrocyte sedimentation rate; WBC: white blood cell count; HPF: High Power Field; anti-dsDNA; anti-double-stranded DNA; C3: complement 3; C4: complement 4; eGFR: estimated glomerular filtration rate; ALT: glutamic-pyruvic transaminase; AST glutamic oxalacetic transaminase.

**Table 3:** Association between serum miR-130b-3p and various parameters of early stage LN patients.

Spearman’s rho variable	Relativeexpression ofmiR-130b-3p	
	R	P value
Mean artery pressure(MAP)	0.182	0.261
Serum WBC	−0.038	0.816
Hemoglobin(Hb)	0.134	0.409
Platelets(PLT)	0.097	0.550
Urinary WBC	−0.057	0.725
Urinary RBC	0.001	0.997
rSLEDAI	0.263	0.102
SLEDAI	0.243	0.131
Serum creatinine(SCr)	−0.168	0.299
Blood Urea Nitrogen(BUN)	−0.192	0.236
Serum Uric Acid (UA)	0.178	0.272
C-reaction protein (CRP)	−0.152	0.348
Erythrocyte sedimentation rate(ESR)	0.181	0.264
Serum albumin (ALB)	−0.187	0.247
Glutamic pyruvic transaminase (ALT)	0.131	0.422
Glutamicoxalacetictransaminase (AST)	0.138	0.395
Triglyceride (TG)	0.376	0.017
Total cholesterol (TC)	0.258	0.108
High density lipoprotein(HDL)	−0.106	0.516
Low density lipoprotein (LDL)	0.271	0.091
Activity indices (AI)	−0.223	0.213

Abbreviations: r: Spearman’s rho correlation coefficient; rSLEDAI: Renal related systemic Lupus Erythematosus Disease Activity Index;anti-dsDNA: anti-double-stranded DNA;C3: complement 3; C4: complement 4.
