# Supplementary material for: Up-regulation of Serum MiR-130b-3p Level is Associated with Renal Damage in Early Lupus Nephritis
Source: Sci Rep. 2015 Aug 28;5:12644. doi: 10.1038/srep12644 (PMC4551961; doi:10.1038/srep12644)
Supplement: Supplementary Information [file srep12644-s1.pdf]

Upregulation of Serum MiR-130b-3p Level is Associated with Renal Damage in Early Lupus Nephritis

Authors: Wanpeng Wang, Shan Mou, Ling Wang, Minfang Zhang, Xinghua Shao, Wei Fang, Renhua Lu, Chaojun Qi, Zhuping Fan, Qin Cao, Qin Wang, Yan Fang, Zhaohui Ni*

Supplementary materials

Table 1a: Expression profile of circulating miRNA from early LN patients versus healthy controls

Number	MicroRNA Name	Fold Change	P value
Up-regulated：			
A02	hsa-let-7c-5p	2.4902	0.1951
A11	hsa-miR-4291	2.2638	0.1462
A21	hsa-miR-628-3p	1.8041	0.0235
B02	hsa-miR-199b-5p	2.0192	0.1494
C20	hsa-miR-3135b	2.0052	0.1483
D08	hsa-miR-143-3p	2.0052	0.0738
D12	hsa-miR-29b-3p	2.094	0.2477
D19	hsa-miR-503-5p	2.1641	0.125
E05	hsa-miR-1539	2.094	0.0411
E21	hsa-miR-378a-3p	2.0687	0.1083
E23	hsa-miR-107	2.1269	0.1178
F06	hsa-miR-877-3p	2.3114	0.0566
F07	hsa-miR-4732-3p	2.0017	0.1013
G07	hsa-miR-18a-3p	2.2638	0.0207
G14	hsa-miR-7-5p	2.578	0.0531

G22	hsa-let-7e-5p	2.0867	0.2089
H07	hsa-miR-4301	2.4263	0.0657
I09	hsa-miR-140-5p	2.5337	0.2068
I11	hsa-miR-328-3p	2.0687	0.4031
I12	hsa-miR-652-3p	2.1678	0.4964
J04	hsa-miR-433-3p	2.5293	0.1165
J11	hsa-miR-130b-3p	2.3764	0.0207
J24	hsa-miR-361-5p	2.1829	0.2127
K09	hsa-miR-378e	2.0157	0.0472
K12	hsa-miR-374a-5p	2.5602	0.1093
L11	hsa-let-7f-5p	2.1306	0.2652
L14	hsa-miR-26b-5p	2.094	0.2848
M22	hsa-miR-1260b	2.2599	0.0301
N01	hsa-miR-223-3p	2.0976	0.3597
N16	hsa-miR-142-3p	3.2801	0.2007
N19	hsa-miR-1307-3p	2.3929	0.2232
O02	hsa-miR-425-3p	3.196	0.1161
P04	hsa-miR-145-5p	2.0367	0.3923
P05	hsa-miR-1233-3p	2.1269	0.0192
Down-regulated：			
D04	hsa-miR-3907	-2.3559	0.1699
F08	hsa-miR-22-3p	-2.473	0.4347
F09	hsa-miR-125b-5p	-2.2638	0.3101
G05	hsa-miR-100-5p	-2.364	0.1304
J01	hsa-miR-122-5p	-2.094	0.4255

Table 1b：Expression profile of circulating miRNA from late LN patients versus healthy controls

Number	MicroRNA Name	Fold Change	P value
Down-regulated：			
A14	hsa-miR-1587	-2.1067	0.234
B01	hsa-miR-3610	-2.0777	0.1881
B11	hsa-miR-151b	-4.1339	0.0079
B12	hsa-miR-423-5p	-2.7895	0.068
B18	hsa-miR-21-5p	-2.0777	0.1321
C03	hsa-miR-146a-5p	-6.6116	0.0373
C06	hsa-let-7g-5p	-2.7274	0.0171
C09	hsa-miR-3200-5p	-2	0.1194
C12	hsa-miR-29c-3p	-3.2773	0.0837
D04	hsa-miR-3907	-4.6995	0.0112
D11	hsa-miR-451a	-3.2546	0.0239
D13	hsa-miR-194-5p	-2.3991	0.3339
D15	hsa-miR-30b-5p	-4.1125	0.0607
D17	hsa-miR-125a-5p	-2.7943	0.1899
E07	hsa-miR-130a-3p	-2.8382	0.1865
E12	hsa-miR-29a-3p	-2.3054	0.1376
E15	hsa-miR-191-5p	-6.6807	0.0374
E17	hsa-let-7b-5p	-2.1547	0.2178
E18	hsa-miR-103a-3p	-3.1059	0.1181
F01	hsa-miR-484	-3.1656	0.0842
F04	hsa-miR-423-3p	-2.3214	0.0517
F08	hsa-miR-22-3p	-6.6576	0.029
F09	hsa-miR-125b-5p	-5.4736	0.1556
F19	hsa-miR-186-5p	-2.9949	0.0461
F22	hsa-miR-1280	-2.1398	0.0937
G04	hsa-miR-148b-3p	-2.775	0.131
G05	hsa-miR-100-5p	-4.9075	0.0646

G20	hsa-miR-30d-5p	-7.1231	0.0211
H05	hsa-miR-199b-3p	-2.1924	0.1857
H16	hsa-miR-221-3p	-3.7581	0.0994
H17	hsa-miR-28-3p	-2.1924	0.1898
H24	hsa-miR-26a-5p	-2.5624	0.0494
I02	hsa-miR-144-3p	-4.7733	0.0664
I03	hsa-miR-20a-5p	-3.4641	0.0201
I04	hsa-miR-195-5p	-3.1113	0.1615
I10	hsa-let-7d-3p	-2.1473	0.1609
I15	hsa-miR-27a-3p	-2.2191	0.0526
I23	hsa-miR-23b-3p	-4.3319	0.1692
J01	hsa-miR-122-5p	-5.8971	0.3286
J05	hsa-miR-140-3p	-2.918	0.1146
J07	hsa-miR-24-3p	-5.0894	0.0216
J14	hsa-miR-4505	-2.1287	0.1446
J17	hsa-miR-1247-5p	-2.166	0.0939
J21	hsa-miR-148a-3p	-3.3694	0.0462
K02	hsa-miR-30c-5p	-2.7798	0.1543
K06	hsa-miR-192-5p	-2.5315	0.3345
K15	hsa-miR-342-3p	-3.4822	0.0193
K19	hsa-miR-27b-3p	-2.9485	0.1982
K20	hsa-miR-15b-5p	-2.8138	0.0581
K22	hsa-miR-373-5p	-4.4	0.0259
L01	hsa-miR-574-3p	-2.3661	0.2167
L04	hsa-miR-17-5p	-3.7321	0.0699
L23	hsa-miR-23a-3p	-4.8065	0.0305
M06	hsa-miR-146b-5p	-2.1251	0.0815
M07	hsa-miR-744-5p	-3.3058	0.1064
M09	hsa-miR-16-5p	-3.2322	0.1086
M13	hsa-miR-106b-5p	-4.5237	0.0335
M23	hsa-miR-126-3p	-2.1104	0.0783

N01	hsa-miR-223-3p	-3.323	0.0644
N03	hsa-miR-25-3p	-2.8629	0.1315
N05	hsa-miR-19a-3p	-6.498	0.0252
N07	hsa-miR-151a-5p	-2.4284	0.2305
N08	hsa-miR-320a	-3.7908	0.0029
N11	hsa-miR-30a-5p	-3.6238	0.1022
N15	hsa-miR-19b-3p	-5.9484	0.0215
N17	hsa-miR-30e-5p	-6.1582	0.1033
N22	hsa-miR-20b-5p	-3.3115	0.0837
O08	hsa-miR-486-5p	-2.853	0.5616
O13	hsa-miR-93-5p	-5.0982	0.0541
O15	hsa-miR-425-5p	-5.063	0.0519
O17	hsa-miR-92a-3p	-2.3295	0.3416
O18	hsa-miR-222-3p	-2.0777	0.2397
O20	hsa-miR-365b-3p	-2.4751	0.0738
P11	hsa-miR-197-3p	-4.2208	0.0121
P12	hsa-miR-126-5p	-2.3335	0.066

Table 1c：Expression profile of circulating miRNA from late LN patients versus early LN patients

Number	MicroRNA Name	Fold Change	P value
Down-regulated：			
A02	hsa-let-7c-5p	-3.2238	0.01
A11	hsa-miR-4291	-2.7062	0.1272
B01	hsa-miR-3610	-2.0438	0.2159
B11	hsa-miR-151b	-3.6395	0.0328
B12	hsa-miR-423-5p	-2.946	0.1342
B14	hsa-miR-93-3p	-2.0332	0.113
B17	hsa-miR-150-5p	-2.2326	0.1506
B18	hsa-miR-21-5p	-3.235	0.0141
B24	hsa-miR-193a-5p	-1.8452	0.0358
C03	hsa-miR-146a-5p	-9.3746	0
C06	hsa-let-7g-5p	-3.3607	0.1396
C12	hsa-miR-29c-3p	-3.2071	0.1466
C16	hsa-miR-181b-5p	-2.1678	0.1168
C24	hsa-miR-324-5p	-2.2835	0.0488
D11	hsa-miR-451a	-4.381	0.001
D13	hsa-miR-194-5p	-3.2518	0.1417
D15	hsa-miR-30b-5p	-4.4268	0.1212
D17	hsa-miR-125a-5p	-2.5293	0.2601
E07	hsa-miR-130a-3p	-5.1204	0.0109
E09	hsa-miR-339-5p	-3.114	0.0426
E12	hsa-miR-29a-3p	-2.6875	0.1505
E15	hsa-miR-191-5p	-9.489	0.1209
E17	hsa-let-7b-5p	-3.9621	0.0082
E18	hsa-miR-103a-3p	-6.0892	0.0622
F01	hsa-miR-484	-3.2575	0.0323
F04	hsa-miR-423-3p	-2.473	0.1322
F08	hsa-miR-22-3p	-2.6921	0.1976

F09	hsa-miR-125b-5p	-2.4179	0.1117
F11	hsa-let-7i-5p	-2.8704	0.0172
F19	hsa-miR-186-5p	-3.6019	0.0517
G01	hsa-miR-151a-3p	-2.5646	0.046
G04	hsa-miR-148b-3p	-3.8271	0.0023
G05	hsa-miR-100-5p	-2.0759	0.0262
G07	hsa-miR-18a-3p	-1.9036	0.026
G14	hsa-miR-7-5p	-2.6459	0.043
G20	hsa-miR-30d-5p	-6.7681	0.0162
G22	hsa-let-7e-5p	-2.8506	0.184
G24	hsa-miR-345-5p	-1.9403	0.0308
H05	hsa-miR-199b-3p	-3.9279	0.0162
H07	hsa-miR-4301	-2.0687	0.0708
H13	hsa-miR-1301-3p	-2.3477	0.0415
H16	hsa-miR-221-3p	-4.6388	0.0049
H17	hsa-miR-28-3p	-2.0473	0.1307
H24	hsa-miR-26a-5p	-3.8337	0.0606
I02	hsa-miR-144-3p	-4.4114	0.0902
I03	hsa-miR-20a-5p	-3.7289	0.0158
I04	hsa-miR-195-5p	-5.3194	0.0083
I06	hsa-miR-4454	-2.5869	0.0347
I09	hsa-miR-140-5p	-2.1306	0.2365
I12	hsa-miR-652-3p	-3.3958	0.1212
I15	hsa-miR-27a-3p	-2.4095	0.1045
I17	hsa-miR-185-5p	-2.7919	0.0583
I23	hsa-miR-23b-3p	-4.188	0.0355
J01	hsa-miR-122-5p	-2.8162	0.3306
J04	hsa-miR-433-3p	-1.8009	0.0127
J05	hsa-miR-140-3p	-2.6413	0.0774
J07	hsa-miR-24-3p	-7.5488	0.085
J08	hsa-let-7d-5p	-2.6095	0.0666

J11	hsa-miR-130b-3p	-2.3114	0.0236
J13	hsa-let-7a-5p	-2.6505	0.1277
J21	hsa-miR-148a-3p	-4.0523	0.0449
J24	hsa-miR-361-5p	-2.094	0.2173
K02	hsa-miR-30c-5p	-2.6049	0.1783
K06	hsa-miR-192-5p	-2.2019	0.1741
K12	hsa-miR-374a-5p	-2.9665	0.0422
K15	hsa-miR-342-3p	-4.9977	0.007
K19	hsa-miR-27b-3p	-4.2908	0.0315
K20	hsa-miR-15b-5p	-4.8442	0.0431
K22	hsa-miR-373-5p	-2.5162	0.3676
K23	hsa-miR-374c-5p	-2.2796	0.1697
K24	hsa-miR-375	-2.9768	0.1625
L01	hsa-miR-574-3p	-2.7582	0.0733
L04	hsa-miR-17-5p	-5.0237	0.0139
L11	hsa-let-7f-5p	-3.2972	0.1748
L14	hsa-miR-26b-5p	-4.1807	0.15
L23	hsa-miR-23a-3p	-5.4311	0.044
M06	hsa-miR-146b-5p	-3.3607	0.0023
M07	hsa-miR-744-5p	-5.3749	0.0251
M09	hsa-miR-16-5p	-5.0237	0.0047
M13	hsa-miR-106b-5p	-6.3149	0.0343
M17	hsa-miR-320e	-1.8709	0.0498
M22	hsa-miR-1260b	-3.7289	0.0048
M23	hsa-miR-126-3p	-2.4137	0.0618
N01	hsa-miR-223-3p	-6.9704	0.2649
N03	hsa-miR-25-3p	-3.1629	0.0868
N05	hsa-miR-19a-3p	-7.1046	0.006
N07	hsa-miR-151a-5p	-2.3681	0.1092
N08	hsa-miR-320a	-3.114	0.0293
N10	hsa-miR-15b-3p	-1.5815	0.0473

N11	hsa-miR-30a-5p	-4.7199	0.0161
N15	hsa-miR-19b-3p	-8.5965	0.0266
N16	hsa-miR-142-3p	-4.6549	0.1894
N17	hsa-miR-30e-5p	-7.0555	0.0052
N19	hsa-miR-1307-3p	-2.0509	0.108
N22	hsa-miR-20b-5p	-3.2858	0.0133
O02	hsa-miR-425-3p	-2.3929	0.2369
O05	hsa-miR-10b-5p	-1.8229	0.0482
O08	hsa-miR-486-5p	-2.3436	0.6779
O13	hsa-miR-93-5p	-5.6716	0.0049
O15	hsa-miR-425-5p	-4.5908	0.016
O17	hsa-miR-92a-3p	-2.3722	0.3102
O18	hsa-miR-222-3p	-3.7289	0.051
O20	hsa-miR-365b-3p	-2.725	0.3096
P01	hsa-miR-18a-5p	-2.4095	0.1023
P04	hsa-miR-145-5p	-3.0764	0.2097
P11	hsa-miR-197-3p	-4.2834	0.0666
P12	hsa-miR-126-5p	-2.1716	0.001

Table 2 MicroRNA Target Prediction Tools

**Name**	**Web SitesAddress**
DIANAmT	http://diana.cslab.ece.ntua.gr/microT
miRanda	http://www.microRNA.org
miRDB	http://mirdb.org/miRDB
miRWalk	http://www.umm.uni-heidelberg.de/apps/zmf/mirwalk/index.html
RNAhybrid	http://bibiserv.techfak.uni-bielefeld.de/rnahybrid
PICTAR	http://pictar.mdc-berlin.de/cgi-bin/new_PicTar_vertebrate.cgi
PITA	http://genie.weizmann.ac.il/pubs/mir07/mir07_data.html
RNA22	https://cm.jefferson.edu/rna22v2
Targetscan	http://www.targetscan.org
